# Increasing prevalence 2015–2019 of amyotrophic lateral sclerosis in Sardinia, Italy

**DOI:** 10.1007/s10072-023-06753-5

**Published:** 2023-03-24

**Authors:** Maria Ida Pateri, Silvy Pilotto, Giuseppe Borghero, Francesca Pili, Vincenzo Pierri, Tommaso Ercoli, Angelo Fabio Gigante, Antonella Muroni, Giovanni Defazio

**Affiliations:** 1 Institute of Neurology, University Hospital of Cagliari, Cagliari, Italy; 2 Department of Medical Sciences and Public Health, University of Cagliari, Cagliari, Italy; 3 Section of Neurology, San Paolo Hospital, Bari, Italy; 4 Amyotrophic Lateral Sclerosis Centre, University of Cagliari, Cagliari, Italy

**Keywords:** Amyotrophic lateral sclerosis, Motor neuron disease, Prevalence, Epidemiology, Sardinia

## Abstract

**Background:**

While amyotrophic lateral sclerosis (ALS) incidence has increased during the last decades, structured evidence on increased prevalence is lacking. After reporting a significant yearly increase of ALS incidence over a 10-year period, we checked for increased prevalence in Southern Sardinia over a quinquennium.

**Methods:**

ALS patients (El Escorial Criteria) recruited from the study area and followed at ALS Centre, University of Cagliari, were included. Prevalence was computed for January 1, 2015 and January 1, 2019 and was calculated for the overall ALS population as well as for tracheostomized and non-tracheostomized patients.

**Results:**

We observed a non-significant trend for greater ALS prevalence in 2019 than in 2015 (18.31 per 100,000 vs. 15.26 per 100,000; rate ratio: 1.83, *p* = 0.01). By contrast, a significantly raising 2015 to 2019 ALS prevalence was observed in tracheostomized patients. No significant difference could be detected in non-tracheostomized.

**Conclusions:**

We provided the highest prevalence rate to date reported in the worldwide literature, and also showed a non-significant raising ALS prevalence in the Sardinian population over a quinquennium. The trend in raising ALS prevalence was likely due to extended survival due to invasive interventions.

## Introduction

Amyotrophic lateral sclerosis (ALS) is generally regarded as a rare disease characterized by heterogeneous motor phenotype, variable course, and fatal prognosis [1, 2]. Worldwide prevalence of ALS ranges between 3.4 and 12.3 per 100,000 persons, with higher estimates in Europe [3–8].

In Sardinia, the last ALS crude prevalence estimation was 10.8 per 100,000 (95% CI: 8.6 to 13.1) on December 31, 2009 [8]. We recently reported a significant yearly increase of ALS incidence in Southern Sardinia over the 10-year period 2010–2019 [9]. In the present study, we therefore analyzed ALS prevalence on two different dates over a quinquennium (January 1, 2015, and January 1, 2019) in the same area and using the same methodology of the incidence study.

## Methods

The study area included 3 of the 5 administrative Sardinian subdivisions (the provinces of Cagliari, South Sardinia, and Oristano), covered 9768 km^2^ (about one-half of the whole island), and hosted nearly two-thirds of the whole Sardinian population. Because the borders of the major administrative Sardinian subdivisions changed widely over the last 15 years, we considered the people of all ages who resided in the 211 municipalities included in the three provinces in 2019 and computed the correspondent population on the two prevalence dates. On January 1, 2015, there were 963,329 individuals (465,744 men and 497,585 women), of whom 207,377 [21.5%] aged 65 or more; on January 1, 2019, there were 928.460 individuals (455.282 men and 472.818 women), of whom 225.814 (24.3%) aged 65 or more.

The ascertainment methodology has been previously described [9]. In brief, the medical facilities for ALS across the study area included one tertiary referral Centre located at the Institute of Neurology of the University of Cagliari, a genetic point reference, seven general neurology clinics, and several field neurologists. All facilities were part of the National Health System Network with free access for Sardinian population. Whatever the initial referral, patients were usually followed up at the University Centre. This Centre served as referral even for patients living outside the study area. Patients that did not reside in the study area were excluded from analysis. In the University Centre, medical records were checked for duplicate and examined to fill in a standardized form containing data on age, sex, age at diagnosis, diagnostic delay (time elapsing between onset of symptoms and diagnosis), clinical phenotype, family history of ALS/dementia/parkinsonism, presence of C9orf72 and TARDBP mutations, the most frequent causative ALS mutations in Sardinia [10], percutaneous endoscopic gastrostomy (PEG), and invasive mechanical ventilation via tracheostomy. A prevalent case was defined as any individual fulfilling El Escorial revised diagnostic criteria [11] who was alive on prevalence date and resided in the study area. Data were collected over the previous 12 years for the 2015 prevalent cases, and over the previous 14 years for the 2019 prevalent cases [11].

Data were expressed as mean and standard deviations (SD) unless otherwise indicated. Statistical analysis was performed by STATA [11] package. Differences between groups were tested by the Mann–Whitney *U* test, the chi-square test, and the Fisher test as appropriate. ALS prevalence rates and 95% confidence intervals (CI) were computed based on the number of ALS patients who were alive on the prevalence date (the numerator) and the general population (the denominator) obtained by ISTAT census data (www. istat.it). Rate ratio was computed to compare rates from the two prevalence days. The study was approved by the local Institutional Review Board.

## Results

The two prevalent groups did not differ for most demographic, clinical, and genetic features (Table 1). However, cases from January 1, 2019, showed a significantly lower age at ALS onset, a significantly higher frequency of subjects aged < 65 years at prevalence date, and a greater frequency of tracheostomy. Most tracheostomized patients (19/26 in the 2015 group and 37/46 in the 2019 group, *p* = 0.47) also underwent PEG.

**Table 1:** Demographic and clinical features of amyotrophic lateral sclerosis prevalent cases on January 1 2015 and January 1 2019

	January 1 2015 (n. 147)	January 1 2019 (n. 170)	*p*
Sex (men/women)	93/54	103/67	0.9
Mean age at diagnosis (years) ± SD	64.4 ± 11.9	61.6 ± 11.4	0.02
N. patients aged 65 or more at the prevalence date	103 (70%)	97 (53%)	0.02
Mean diagnostic delay (months) ± SD	18.3 + 18.5	16.7 + 15.1	0.2
Clinical phenotype at onset			
* Classic limb onset*	65	66	0.33
* Bulbar onset*	25	28	0.89
* Flail arm*	18	27	0.35
* Flail leg*	28	24	0.24
* Predominantly upper motor neuron*	8	15	0.25
* Pure upper motoneuron*	0	0	NA
* Pure lower motoneuron*	2	3	0.77
* Respiratory*	1	3	0.63
* Unknown*	0	4	0.1
Family history of			
* Amyotrophic lateral sclerosis*	24	36	0.27
* Dementia/parkinsonism alone*	13	21	0.36
Genetic analysis			
* C9orf72*	10	18	0.45
* TARDPB*	44	55	
* C9orf72 and/or TARDPB*	3	3	
Tracheostomy at prevalence date	26	46	0.047

The overall prevalence rates were 15.26 per 100,000 on January 1, 2015, and 18.31 per 100,000 on January 1, 2019 (Table 2). Rate ratio indicated a non-significant trend for greater ALS prevalence in 2019, and a significantly raising 2015 to 2019 ALS prevalence in tracheostomized patients (Table 2). The finding was also observed when we analyzed only the smaller sample of patients undergoing both tracheostomy and PEG (prevalence rate: 1.97 per 100,000 vs. 3.98 100,000; rate ratio: 2.02, *p* = 0.01).

**Table 2:** Prevalence estimates on January 1 2015 and January 1 2019

	Prevalence on January 1 2015	Prevalence on January 1 2019	Rate ratio (2019/2015) (95% confidence interval), *p*
*Overall population*			
*Study population* (*n*)	963,329	928,460	1.20 (0.96–1.51), 0.11
*Prevalent cases* (*n*)	147	170	
*Prevalence rate* (× *100,000 persons*) [*95% confidence interval*]	15.26 (12.89–17.94)	18.31 (15.66–21.28)	
*People undergoing tracheostomy at prevalence date*			
*Study population* (*n*)	963,329	928,460	1.83 (1.11–3.09), 0.012
*Prevalent cases* (*n*)	26	46	
*Prevalence rate* (× *100,000 persons*) [*95% confidence interval*]	2.69 (1.76–3.95)	4.95 (3.63–6.61)	
*People who were not tracheostomized at prevalence date*			
*Study population* (*n*)	963,329	928,460	1.06 (0.82–1.38), 0.63
*Prevalent cases* (*n*)	121	124	
*Prevalence rate* (× *100,000 persons*) [*95% confidence interval*]	12.56 (10.42–15.01)	13.36 (11.11–15.92)	

Demographic and clinical data of non-tracheostomized patients are reported in Table 3. In this subgroup, prevalence estimates consistently decreased on both prevalence days but no significant difference could be detected by rate ratios, even when we stratified by age, either sex, or patients undergoing PEG alone (Table 4).

**Table 3:** Demographic and clinical features of non-tracheostomized prevalent cases on January 1 2015 and January 1 2019

	Prevalent cases on January 1 2015 (n.121)	Prevalent cases on January 1 2019 (n.124)	*p*
Sex (men/women)	77/44	76/48	0.8
Mean age at diagnosis (years) + SD	65.2 ± 12.5	61.9 ± 11.1	0.02
N. patients aged 65 or more at the prevalence date (%)	76 (62%)	97 (53%)	0.01
Mean diagnostic delay (months) + SD	20.5 ± 19.8	18.5 ± 16.4	0.2
Clinical phenotype at onset			
* Classic limb onset*	47	50	0.81
* Bulbar onset*	22	20	0.61
* Flail arm*	16	22	0.33
* Flail leg*	26	15	0.05
* Predominantly upper motor neuron*	8	13	0.31
* Pure upper motoneuron*	0	0	NA
* Pure lower motoneuron*	2	2	0.96
* Respiratory*	0	2	0.17
* Unknown*	0	0	NA
Family history of			
* Amyotrophic lateral sclerosis*	22	28	0.27
* Dementia/parkinsonism alone*	12	15	0.36
Genetic analysis			
C9orf72	6	8	0.45
TARDPB	40	28	
C9orf72 and/or TARDPB	3	2	
PEG at prevalence date	8	12	0.4

**Table 4:** Prevalence estimates in patients who were not tracheostomized at the prevalence date

*People who did not undergo tracheostomy at prevalence date*	Prevalence on January 1 2015	Prevalence on January 1 2019	Rate ratio 2019/2015 (95% confidence interval), *p*
*Overall population*			
*Study population* (*n*)	963,329	928,460	1.09 (0.84–1.41), 0.50
*Prevalent cases* (*n*)	121	124	
*Prevalence rate* (× *100,000 person*) [*95% confidence interval*]	12.56 (10.42–15.01)	13.36 (11.11–15.92)	
*Women*			
*Study population* (*n*) [%]	497,585	472,818	1.15 (0.75–1.77), 0.51
*Prevalent cases* (*n*)	44	48	
*Prevalence rate* (× *100,000 person*) [*95% confidence interval*]	8.84 (6.42–11.87)	10.15 (7.49–13.46	
*Men*			
*Study population* (*n*) [%]	465,744	455,282	1.0 (0.73–1.43), 0.95
*Prevalent cases* (*n*)	77	76	
*Prevalence rate* (× *100,000 person*) [*95% confidence interval*]	16.53 (13.05–20.66)	16.69 (13.15–20.89)	
*People aged* ≥ *65 years at prevalence date*			
*Study population* (*n*) [%]	207,377	225,814	0.81 (0.57–1.14), 0.21
*Prevalent cases* (*n*)	76	67	
*Prevalence rate* (× *100,000 person*) [*95% confidence interval*]	36.65 (28.87–45.87)	29.67 (22.99–37.68)	
*People aged* < *65 years at prevalence date*			
*Study population* (*n*) [%]	755,952	702,646	1.32 (0.90–2.06), 0.12
*Prevalent cases* (*n*)	45	57	
*Prevalence rate* (× *100,000 person*) [*95% confidence interval*]	5.95 (4.34–7.96)	8.11 (6.14–10.51)	
*People who undervent PEG alone*			
*Study population* (*n*) [%]	963,329	928,460	1.56 (0.56–0.39), 0.34
*Prevalent cases* (*n*)	8	12	
*Prevalence rate* (× *100,000 person*) [*95% confidence interval*]	0.83 (0.36–1.63)	1.29 (0.67–2.26)	

## Discussion

Our analysis yielded in 2019 the highest ALS crude prevalence estimate to date reported in the worldwide literature. Although the 2019 estimate was greater than the 2015 estimate, rate ratio indicated only a non-significant trend for raising prevalence over the quinquennium. However, when we separately analyzed patients who underwent tracheostomy and those who did not, rate ratio indicated a significantly higher prevalence of tracheostomized ALS patients in 2019. By contrast, the rates excluding tracheostomized patients were very similar and were both in the upper part of the range of variability of the estimates reported in the last 15 years in Italy and other European countries [3, 4, 6, 7, 12–15].

The significant rate ratio associated to invasive interventions like tracheostomy and PEG would probably indicate a major contribution of extended survival due to disease treatment and multidisciplinary management to raising prevalence in this subgroup [16, 17]. To our knowledge, most earlier studies did not provide any data on invasive procedures. Only one recent study by Chiò et al. considered tracheostomized and non-tracheostomized patients in the prevalence calculation [7]. In this study, however, the percentage of tracheostomized patients was lower than in our 2015 and 2019 surveys (14% vs. 20% to 27%). The greater frequency of tracheostomy among our patients may reflect differences in the social and religious attitude between Sardinian and other populations, but the attitude of physicians may also be important. It is worth noting that the earlier study collected information from an ALS registry resulting from the contribution of several physicians that could have variable attitude toward invasive procedures. By contrast, in our setting, only one group faced with ALS patients once diagnosis was made. Indeed, tracheostomy was not provided more easily by the National Health System over the referral years.

This study has strengths and limitations. Incomplete case ascertainment would have occurred but relying on multiple data sources likely limited this bias. In addition, data were collected over sufficiently long time periods (the previous 12 years in the 2015 prevalent cases, the previous 14 years in the 2019 prevalent cases) to fully identify the whole prevalent population, including long-survival patients [13]. A diagnostic bias cannot be excluded, even though diagnostic accuracy was assured by the involvement of neurologists with long standing experience in ALS (GB) [9, 14] who applied the revised El Escorial criteria and included only patients with a definite diagnosis of ALS. Supporting the accuracy and generalizability of findings, our cohorts were similar to the general ALS population for men preponderance, age at onset, frequency of clinical phenotypes, and high rate of family history of ALS and C9orf72/TARDBP mutations [5]. The lack of significant changes in the prevalence estimate of non-tracheostomized ALS patients over the examined quinquennium could also reflect the relatively short study period.

In conclusion, we provided the highest prevalence rate to date reported in the literature, and also showed a non-significant raising ALS prevalence in the Sardinian population over a quinquennium. The trend in raising ALS prevalence was mainly due to the extended survival due to invasive interventions. The rate considering tracheostomy should be considered as a measure of ALS burden for the health and social system, while the rate computed after excluding tracheostomized patients would reflect the disease risk more closely. Our results would be relevant to track the evolving epidemiology of ALS. Owing to extended survival due to disease treatment and multidisciplinary management, it is possible that in the near future ALS should not be considered a rare disease any longer, at least not in some countries (the rare disease threshold varies from 11 of 100,000 inhabitants in Australia to 50 of 100,000 in the European Union and 75 of 100,000 in the USA).

## Data Availability

All data supporting the findings of this study are available within the paper.

## References

[1] Brown RH, Al-Chalabi A (2017). Amyotrophic lateral sclerosis. N Engl J Med.

[2] Belbasis L, Bellou V, Evangelou E (2016). Environmental risk factors and amyotrophic lateral sclerosis: an umbrella review and critical assessment of current evidence from systematic reviews and meta-analyses of observational studies. Neuroepidemiology.

[3] Brown CA, Lally C, Kupelian V, Flanders WD (2021). Estimated Prevalence and Incidence of Amyotrophic Lateral Sclerosis and SOD1 and C9orf72 Genetic Variants. Neuroepidemiology.

[4] Puopolo M, Bacigalupo I, Piscopo P (2021). Latium Registry Group, Vanacore N. Prevalence of amyotrophic lateral sclerosis in Latium region, Italy. Brain Behav.

[5] Borg R, FarrugiaWismayer M (2021). Genetic analysis of ALS cases in the isolated island population of Malta. Eur J Hum Genet.

[6] Xu L, Liu T, Liu L (2020). Global variation in prevalence and incidence of amyotrophic lateral sclerosis: a systematic review and meta-analysis. J Neurol.

[7] Chiò A, Mora G, Moglia C, Manera U, Canosa A, Cammarosano S, Ilardi A, Bertuzzo D, Bersano E, Cugnasco P, Grassano M, Pisano F, Mazzini L, Calvo A, Piemonte and Valle d’Aosta Register for ALS (PARALS) (2017). Secular trends of amyotrophic lateral sclerosis: the Piemonte and Valle d'Aosta Register. JAMA Neurol.

[8] Pugliatti M, Parish LD, Cossu P (2013). Amyotrophic lateral sclerosis in Sardinia, insularItaly, 1995–2009. J Neurol.

[9] Borghero G, Pierri V, Vasta R (2022). Incidence of amyotrophic lateral sclerosis in Sardinia, Italy: age-sex interaction and spatial-temporal variability. Amyotroph Lateral Scler Frontotemporal Degener.

[10] Borghero G, Pugliatti M, Marrosu F (2014). Genetic architecture of ALS in Sardinia. Neurobiol Aging.

[11] Brooks BR, Miller RG, Swash M, Munsat TL (2000). El Escorial revisited: revised criteria for the diagnosis of amyotrophic lateral sclerosis. Amyotroph Lateral Scler Other Motor Neuron Disord.

[12] Palese F, Sartori A, Verriello L (2019). Epidemiology of amyotrophic lateral sclerosis in Friuli-Venezia Giulia, North-Eastern Italy, 2002–2014: a retrospective population-based study. Amyotroph Lateral Scler Frontotemporal Degener.

[13] Scialò C, Novi G, Bandettini di Poggio M (2016). Clinical epidemiology of amyotrophic lateral sclerosis in Liguria, Italy: an update of LIGALS register. Amyotroph Lateral Scler Frontotemporal Degener.

[14] Pugliatti M, Parish LD, Cossu P (2013). Amyotrophic lateral sclerosis in Sardinia, insular Italy, 1995–2009. J Neurol.

[15] Vasta R, Moglia C, Manera U (2022). What is amyotrophic lateral sclerosis prevalence?. Amyotroph Lateral Scler Frontotemporal Degener.

[16] Cetin H, Rath J, Füzi J (2015). Epidemiology of amyotrophic lateral sclerosis and effect of riluzole on disease course. Neuroepidemiology.

[17] Chiò A, Moglia C, Canosa A (2022). Respiratory support in a population-based ALS cohort: demographic, timing and survival determinants. J Neurol Neurosurg Psychiatry.

